# The Emerging Role and Clinical Utility of Transnasal Transesophageal Echocardiography

**DOI:** 10.1111/echo.70511

**Published:** 2026-05-25

**Authors:** Ethan Tumarkin, Satvik Ramakrishna, Libo Wang

**Affiliations:** ^1^ Division of Cardiology, Department of Medicine University of Utah Health Salt Lake City Utah USA

**Keywords:** cardiac hemodynamics, cardiac imaging, transesophageal echo, transnasal

## Abstract

Transesophageal echocardiography (TEE) is a cornerstone of cardiovascular diagnostics. A major advantage of TEE over transthoracic echo (TTE) is superior resolution of posterior structures such as the mitral valve and the left atrial appendage (LAA). TEE is invasive and requires the use of moderate and sometimes deeper levels of sedation. Anesthesia accounts for most TEE‐related risks including hemodynamic (hypotension, arrythmia) and respiratory compromise. Risks of anesthesia are amplified by the high prevalence of co‐morbidities in patients undergoing TEE. Anesthesia also requires specialized procedural space and skilled providers, limiting the availability of TEE, and increasing the overall cost of the procedure. Alternative TEE methods that minimize (or negate) the use of anesthesia would enhance workflow, expand availability, reduce risk, and may provide similar or more accurate information compared to conventional TEE. The nasal orifice is an alternative to the oral approach, and transnasal TEE can eliminate the need for anesthesia and expand availability of use, while minimizing risk of oropharyngeal injury. This review explores the emerging role of transnasal TEE as a viable alternative to the conventional oral approach and critically evaluates current technologies and clinical applications. Particular attention is given to unmet clinical needs, barriers to adoption, and future directions that may enable broader, safer, and more effective use of TEE in both procedural and non‐procedural settings.

## Graphical abstract

Transesophageal echocardiography (TEE) is a cornerstone of cardiovascular diagnostics. Alternative TEE methods that minimize the use of anesthesia would enhance workflow, expand availability, reduce risk, and may provide similar or more accurate information compared to conventional TEE (a). Transnasal TEE (b) can eliminate the need for anesthesia and expand availability of use, while minimizing risk of oropharyngeal injury.

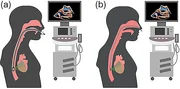

## 1 Introduction

Transesophageal echocardiography (TEE) is an essential diagnostic tool in cardiology [1]. The main advantage over transthoracic echocardiography (TTE) is superior resolution of posterior cardiac structures, including the evaluation of the left atrial appendage (LAA) for thrombus [2, 3, 4] and in assessing the severity of mitral valve regurgitation (MR) [5, 6], given the elimination of imaging artifact from lung scatter which often degrades or obscures TTE images. More recently, the use of TEE has become standard practice in the diagnosis and management of structural heart disease to guide complex minimally invasive transcatheter procedures, owing to enhanced visualization of cardiac structures (in 2D and 3D) and intracardiac instrumentation. TEE has also become the go‐to imaging modality in the critical care setting when TTE imaging is sub‐optimal (recent sternotomy, bandages, pneumomediastinum, etc.) and there is concern for cardiac hemodynamic instability. TEE imaging allows for consistent visualization of cardiac structures and function, but also for assessment of real time hemodynamic parameters. Along these lines, continuous cardiac imaging has been shown to be effective for guiding management in critically ill patients [7]. However, TEE is invasive and requires the use of anesthesia prior to insertion of the TEE probe into the esophagus, which necessitates specialized space and skilled providers, increasing cost, patient risk, and limiting availability [8]. Up to 80% of TEEs results in at least mild oropharyngeal injury, with up to 40% resulting in complex lesions [9, 10]. Anesthesia accounts for most TEE‐related risks including hemodynamic (hypotension, arrythmia) and respiratory compromise; yet is often crucial for procedural success even with adequate topicalization [11, 12, 13]. The serious risks of anesthesia are amplified by the high prevalence of co‐morbidities in patients undergoing TEE, particularly in critically ill patients. Anesthesia‐induced hypotension can also confound the assessment of MR, which is highly sensitive to changes in blood pressure and afterload [14, 15, 16]. Alternative TEE methods that minimize (or negate) the use of anesthesia will enhance workflow, expand availability, reduce risk, and may provide similar or more accurate information compared to conventional TEE. Conversely, intracardiac echo (ICE) probes (inserted into the vascular space for use in invasive cardiac procedures) are only 3–4 mm in diameter; although ICE is not an ideal alternative to TEE due to increased invasiveness (requiring dedicated venous access), the need for specialized procedural space, and a highly skilled procedural team.

The nasal orifice is an alternative to the oral approach (Figure 1), and transnasal TEE, with the appropriate equipment, can eliminate the need for anesthesia, reduce risk of oropharyngeal injury, and allow for physiologic data in the unsedated patient. We define transnasal TEE as the insertion of an ultrasound transducer through the nasal passage into the nasopharynx and subsequently guided into the esophagus or stomach for cardiac visualization. Early investigations relied upon custom‐engineered miniature TEE probes (6.0–7.3 mm diameter). As the technique evolved, subsequent studies established the feasibility of utilizing standard pediatric TEE probes (approximately 7.0–8.0 mm) via a transnasal approach. Building upon these findings, several reports have since documented the ‘off‐label’ use of intracardiac echocardiography (ICE) catheters as de facto TEE probes [17, 18, 19, 20, 21, 22]. A recent comprehensive review delineates the relative strengths and limitations of these various probes within the context of structural heart disease (SHD) guidance [23, 24]. While that review does not specifically address the transnasal TEE approach, it provides a robust framework for understanding the performance characteristics for each probe class. The present discussion builds upon those comparisons by examining the specific utility of these technologies when deployed via the nasal route. Table 1 summarizes key characteristics of discussed cardiac probes, and Figure 2 shows the relative size of a standard TEE, pediatric (micro), and ICE probes.

**FIGURE 1:**
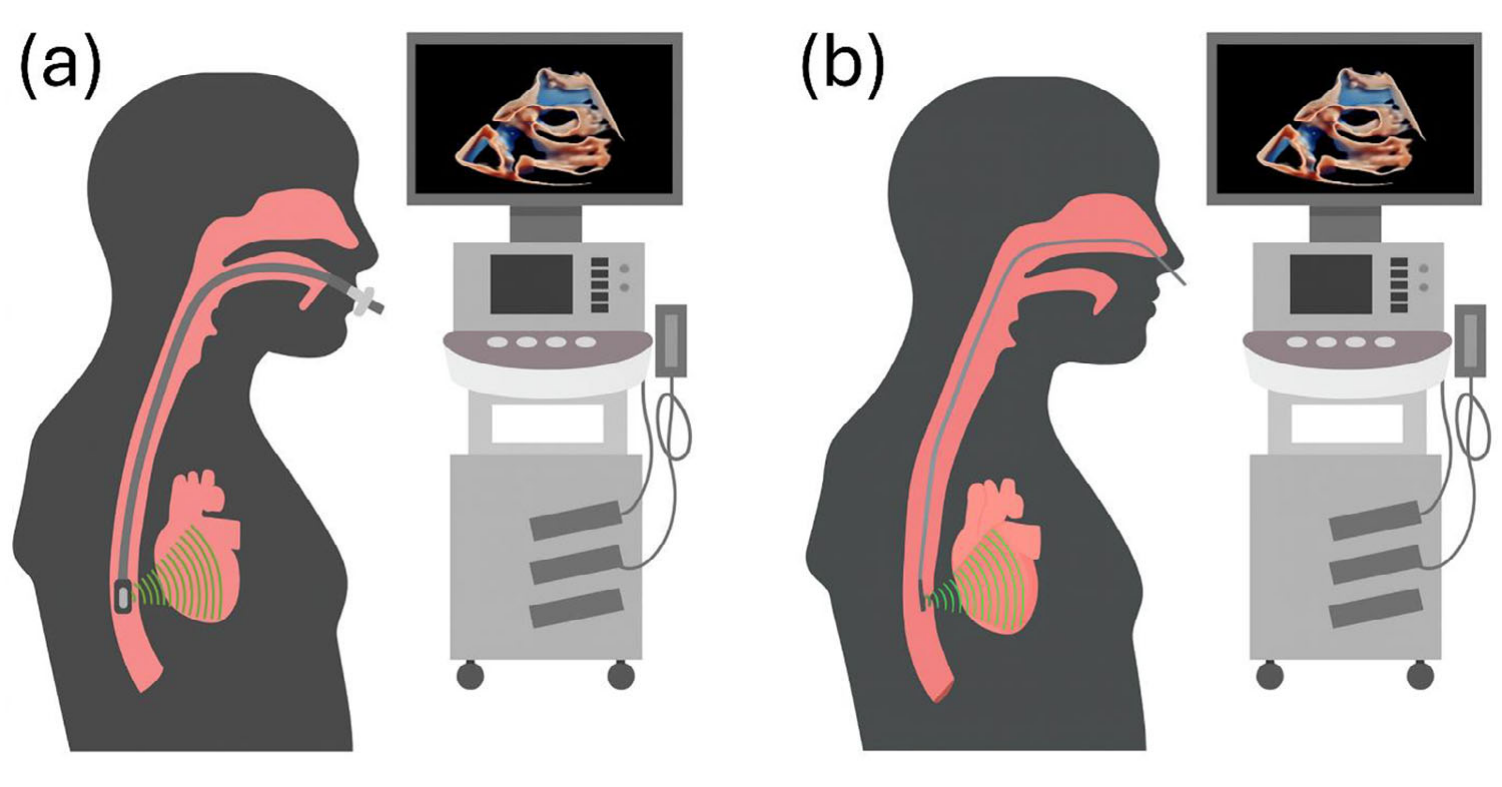
(a) Schematic demonstrating transesophageal echo (transoral), and (b) Transnasal transesophageal echo.

**TABLE 1:** Comparison of the typical characteristics of echocardiography probes.

	Standard TEE	Pediatric (Micro) TEE	Intracardiac Echo (ICE)
**Physical specifications**			
Primary use	Adults (>20–25 kg)	Infants/Children (> 2.5 kg)	Interventional procedures
Anesthesia	GA, DS, MS	GA, DS, MS	LA
Probe diameter	Large (∼10–16 mm)	Medium to Small (∼5–8 mm)	Very Small (2.7–3.3 mm)
# Transducer elements	2500+	32–48	64 (Acunav) vs 840 (Verisight, Nuvision)
**Performance and clinical characteristics**			
Access route	Esophageal (Oral)	Esophageal (Oral)	Vascular (Femoral Vein)
Resolution	Gold standard	Good; smaller field of view	Good near‐field; limited far‐field
Key rdvantage	Comprehensive 3D/4D	Less traumatic; used in small infants	No general anesthesia; operator‐controlled
Key risk	Esophageal injury, aspiration	Probe heating	Vascular injury, pericardial effusion
Transnasal suitability	Too large, not feasible	Multiplane capable, safe (Especially when used with nasal conduit)	Monoplane, lower image quality, good safety profile in limited studies
Cost	Reusable (Moderate cost per use)	Reusable (Moderate cost per use)	High (Single‐use disposable probe)

Abbreviations: DS, Deep Sedation; GA, General Anesthesia; MS, Moderate Sedation.

**FIGURE 2:**
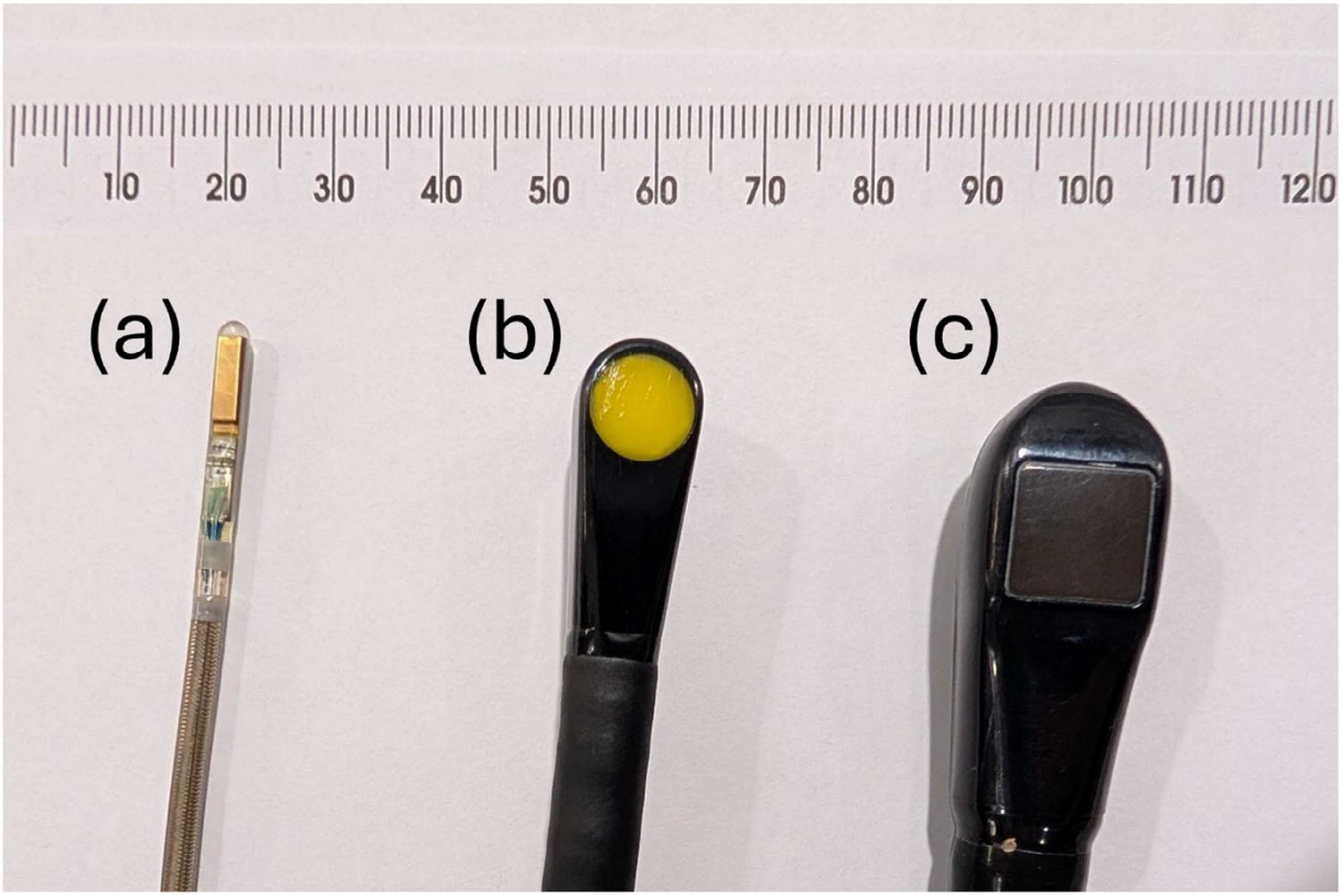
Size comparison of (a) Philips Verisight ICE Probe (3 mm tip width), (b) Philips S8 Pediatric probe (7.5 mm tip width), (c) Philips X8‐2T TEE probe (16.9 mm tip width).

In this review, we highlight the existing literature on transnasal TEE, critically evaluate current technologies and clinical applications, and explore emerging innovations that may further redefine the role of esophageal echocardiography. Particular attention is given to unmet clinical needs, barriers to adoption, and future directions that may enable broader, safer, and more effective use of TEE in both procedural and non‐procedural settings.

## 2 Methods

To conduct this review, we conducted a comprehensive literature search across major medical databases, including PubMed and Scopus, to identify relevant studies on transnasal echocardiography. The search strategy utilized a combination of keywords such as “transnasal echo”, “transnasal echocardiography”, “nasal echo”, “nasal echocardiography”. Peer‐reviewed articles were evaluated by three different clinicians to determine which articles were appropriate for inclusion. The initial search yielded a total of 70 records. After removing 21 duplicates, 49 records remained for title and abstract screening. During the initial screening, 23 articles were excluded as they did not involve transnasal TEE. The remaining 26 articles were assessed for eligibility. A further nine articles were excluded based on predefined criteria: four were editorials or meeting abstracts, two were published in non‐peer‐reviewed journals, two focused on pediatric populations, one was a non‐clinical publication, one article could not be accessed. A total of 17 articles were included in this scoping review. Data was extracted from the articles by the authors and included type of study, size of study, probe utilized (size, and capabilities), success of nasal intubation, patient comfort, adverse reactions, and image quality.

### 2.1 Transnasal TEE Using Mini TEE Probes

The earliest report of transnasal TEE was established by Spencer et al. in 1997 [25]. Their seminal work introduced a newly developed mini monoplane TEE probe with a 32‐element transducer capable of pulse wave and color Doppler. The probe's imaging head measured 7.3 mm in maximal width. This initial study aimed to demonstrate transnasal TEE feasibility (safety/tolerability), adequate image quality, and a new‐use modality as a continuous monitor for LV function (leave‐in’ probe). The study included a comparison arm which utilized a traditional pediatric multiplane TEE probe (9.1 mm width). A total of 128 patients were enrolled, all of whom received local anesthesia to both the nasal cavity and oropharynx. Early in the study, minor mucosal bleeding prompted the investigators to introduce a modified soft plastic nasal trumpet as a conduit for probe insertion; this modification, implemented in the final 37 patients, effectively mitigated further bleeding. Transnasal intubation was generally considered easy, with and without the nasal trumpet, although eight attempts were unsuccessful, most commonly due to difficulty advancing the probe through the proximal nasal passage. Patient tolerance was comparable between transnasal and oral insertion and did not differ significantly between moderate and deep sedation. Image quality of the transnasal probe was considered adequate for assessment of cardiac structure and function but was significantly limited by a lack of multiplane capability. Notably, eight patients in the intensive care unit had the transnasal probe stabilized in the transgastric short‐axis view for up to 2 h for continuous cardiac monitoring. This study was pivotal in demonstrating the feasibility of transnasal TEE. 2 years later, the authors reported similar findings in a larger multicenter cohort using the same custom miniaturized monoplane probe, further reinforcing the feasibility of this approach [26]. In 1998 [27], Spencer and colleagues extended this concept by performing proof‐of‐principle transnasal stress echocardiography in 15 healthy volunteers. Using the same 7.3 mm monoplane probe inserted transnasally via a nasal trumpet. The probe was positioned in the stomach to obtain transgastric LV short‐axis views at the level of the papillary muscles. Participants exercised for a mean duration of 10 ± 2.3 min, with most reporting minimal or mild nasopharyngeal discomfort. Although limited to healthy individuals, this study demonstrated the feasibility of unsedated, exercise‐based transnasal TEE.

In 1999, Greim et al. [28], evaluated the utility of the same miniaturized monoplane probe for transnasal LV assessment in critically ill patients with suspected cardiac dysfunction. In total, 42 mechanically ventilated patients underwent transnasal TEE (with conventional TEE serving as a comparator). Image quality for LV short‐axis and four‐chamber long‐axis views did not differ significantly between transnasal and conventional monoplane TEE. However, the authors concluded that the absence of multiplane transnasal probes limited the role of nasal TEE for comprehensive examinations at that time.

In 2009, Fukuda et al. [29], assessed the feasibility of transnasal TEE for detection of LAA thrombus in patients with atrial fibrillation or prior stroke. A monoplane microprobe (Aloka UST‐5293S; 6.0 mm width) was inserted transnasally without sedation following local nasal anesthesia. Prior to probe insertion, a soft plastic conduit was placed in the nasal cavity. The insertion of the TEE probe was successful in 52 (84%) of 62 patients. Eight patients had unsuitable nasal anatomy, and two were intolerant of probe insertion. Despite 56% of patients receiving warfarin therapy, only two cases of mild, self‐limiting epistaxis were observed. Patient‐reported tolerability was high, with 72% reporting no discomfort during nasal insertion. Transnasal TEE identified a potential intracardiac embolic source in 14 patients, including LAA thrombus (*n* = 10), spontaneous echocardiographic contrast (*n* = 2), patent foramen ovale (PFO; *n* = 1), and mobile aortic plaque (*n* = 1). Limitations included the absence of a conventional multiplane TEE comparator and reliance on early‐generation monoplane technology.

Early mini‐TEE probes were characterized by several inherent technical limitations that constrained their diagnostic utility. These included a reduced element count, and monoplane imaging capabilities that precluded multiplane assessment of complex cardiac structures. Furthermore, these early‐generation probes lacked three‐dimensional (3D) reconstruction capabilities. Despite these hardware restrictions, the clinical application of mini‐TEE represented a significant procedural milestone, establishing feasibility of performing diagnostic TEE in completely unsedated patients. Literatures describing the use of mini‐TEE probes for transnasal TEE are summarized in Table 2.

**TABLE 2:** Summary of publications of transnasal TEE using mini TEE probes.

Author	Year	Study Type	No.	Probe (head width)	Plane View	Type of Anesthesia	Use of Nasal Trumpet/Conduit	Success of Nasal Intubation	Adverse Outcomes	Patient Comfort	Image Quality (Rated by Clinicians)
Spencer et al. [25].	1997	Prospective Study	128	HP mini TEE (7.3 mm)	Mono	Local nare/oropharynx and conscious sedation	Yes (for the last 37 patients)	81% (88% when using the nasal conduit)	1.6% (1 patient) with epistaxis requiring intervention	84% Good, 11% acceptable, 5% poor	Acceptable/good in all cases
Spencer et al. [27].	1998	Prospective Study	15	HP mini TEE (7.3 mm)	Mono	Local nare	Yes	87%	None	85% minimal to mild discomfort	Acceptable/good in all cases
Spencer et al. [26].	1999	Prospective Study	194	HP mini TEE (7.3 mm)	Mono	Local nare/oropharynx ± conscious sedation	Yes	88%	12% mild epistaxis	96% excellent/good, 4% poor	97% acceptable
Greim et al. [28].	1999	Prospective Study	42	HP mini TEE (7.3 mm)	Mono	Local nare/oropharynx ± conscious sedation	No	90%	31% epistaxis, 1 patient required treatment	Overall good, but patients sedated and intubated	Adequate but limited compared to multiplane probe
Greim et al. [30].	2001	Prospective Study	57	HP mini TEE (7.3 mm)	Mono	Local nare/oropharynx + conscious sedation	No	98%	None	Overall good	Acceptable/good in all cases
Zimmerman et al. [31].	2003	Prospective Study	45	HP mini TEE (7.3 mm)	Mono	Local nare/oropharynx ± conscious sedation	No	96%	None	Overall good (1 aborted due to discomfort)	73% good, 23% moderate
Fukuda et al. [29].	2009	Prospective Study	62	Aloka UST‐5293S (6.0 mm)	Mono	Local nare/oropharynx	Yes	84%	3% (2 patients) mild epistaxis	72% no pain/discomfort	Not quantified

### 2.2 Transnasal TEE Using Pediatric TEE Probes

The transition to pediatric micro‐TEE probes represented an important technological advancement in transnasal imaging. While these transducers typically maintained a similar element density to their “mini‐probe” predecessors (ranging from 32–48 elements), they introduced multiplane imaging capabilities. Although these probes remained limited by a lack of volumetric (3D) reconstruction, the integration of an adjustable imaging matrix permitted a more comprehensive assessment of complex cardiac morphology. This shift from monoplane to multiplane arrays significantly enhanced the clinical utility of transnasal TEE by allowing for standard cross sectional views and more precise valvular and structural characterization. Literatures describing the use of pediatric TEE probes for transnasal TEE are summarized in Table 3.

**TABLE 3:** Summary of publications of transnasal TEE using pediatric (Micro) TEE probes.

Author	Year	Study type	No.	Probe (head width)	Plane view	Type of anesthesia	Use of nasal trumpet/ Conduit	Success of nasal intubation	Adverse outcomes	Patient comfort	Image quality (Rated by clinicians)
Klettas et al. [32].	2017	Prospective study	24	Philips S8‐3T (7.5 mm)	Multiplane	Local nare/oropharynx ± conscious sedation	Yes	100%	8% (2 patients) hypoxia (secondary to sedation)	100% good	Good to very good in all cases
Wang et al. [35].	2019	Case report	1	Philips S8‐3T (7.5 mm)	Multiplane	Unclear	No	100% (single case)	None	Well tolerated	Good
Sun et al. [33].	2020	Prospective study	34	Philips S8‐3T (7.5 mm)	Multiplane	Local nare	No	100%	7% (2 patients) mild epistaxis	Well tolerated	Good (66.7%) and acceptable (33.3%)
Lanki et al. [34].	2024	Retrospective/Prospective study	364	Philips S8‐3T (7.5 mm)	Multiplane	Local nare/oropharynx ± conscious sedation	No	98%	5.9% (10 patients) epistaxis (4 patients required treatment)	Not mentioned	88% good or acceptable, but all cases adequate for study

Klettas et al. [32], were the first to perform transnasal TEE with a multiplane capable probe. They utilized the Philips S8‐3T pediatric micro‐TEE probe. The Philips S8‐3T is a TEE microprobe originally designed for pediatric use, with extended operating frequency range of 3–8 MHz and physical dimensions at the tip width of 7.5 mm. The microprobe has multiplane capability and is equipped with steerable PW Doppler, CW Doppler, color Doppler, M‐mode, and harmonic imaging. The study examined whether transnasal TEE could provide sufficient image quality and patient tolerability to avoid general anesthesia during structural heart interventions. The study included 24 patients who underwent one of a transfemoral aortic valve implantation (TAVI), transfemoral mitral valve implantation (TMVI), atrial septal defect (ASD), or PFO closure. All patients underwent both transnasal TEE as well as conventional TEE. Although overall image quality was inferior to that of standard TEE, likely reflecting the substantial disparity in transducer element count compared with contemporary adult probes, anatomic delineation was sufficient for procedural guidance in all cases. No nasal bleeding was reported. The authors concluded that transnasal TEE represents a promising alternative imaging modality for interventional procedures, particularly as a means to avoid general anesthesia.

Sun et al. [33], subsequently used the same S8‐3T microprobe in 30 patients undergoing non‐sedated LAA occlusion. All participants tolerated the transnasal probe with local anesthesia alone. Two cases of mild, self‐resolving epistaxis were reported. Image quality was rated as good in 66.7% and acceptable in 33.3% of cases by independent echocardiographers. All devices were successfully implanted, and follow‐up TEE at a median of 55 days demonstrated no significant residual leaks or device‐related thrombus.

In the most recent and largest trial to date, Lanki et al. [34], evaluated 336 patients undergoing PFO closure at Helsinki University Hospital from 2003–2021. Conventional TEE under general anesthesia was used until November 2018 (*n* = 167) after which nasal TEE was performed using the Philips S8‐3T (*n* = 169). Among patients undergoing transnasal TEE, epistaxis occurred in nine cases (5.3%), with four patients (2.4%) requiring intervention. One patient experienced prolonged nasal congestion lasting two months. Image quality was rated as acceptable to good in 88% of cases. Direct comparison with conventional TEE was not performed, as image quality, even when suboptimal, was sufficient for procedural success. Overall, transnasal TEE was successfully performed in 97.2% of cases.

### 2.3 Transnasal TEE Using ICE Probes

Intracardiac echocardiography (ICE) was introduced into clinical practice in 2000 (Acuson AcuNav 10F) primarily for endovascular guidance during invasive electrophysiological and structural procedures. These catheters, typically ranging from 8F–10F (2.7–3.3 mm) in diameter, feature a 64‐element phased‐array transducer capable of four‐way steering and full Doppler functionality. Operating at frequencies between 5.5 and 10 MHz, ICE provides a 90° sector view with a penetration depth of up to 16 cm. While ICE facilitates complex structural interventions without the need for general anesthesia, its conventional use is limited by its inherent invasiveness requiring dedicated large‐bore venous access, as well as the necessity for a specialized procedural suite and a highly skilled operator. However, an alternative, less invasive approach to both standard TEE and ICE, is the off‐label use of intracardiac echocardiography (ICE) probes transnasally as a de‐facto TEE probe. Several studies have looked at this and are summarized below. Literatures describing the use of ICE probes for transnasal TEE are summarized in Table 4.

**TABLE 4:** Summary of publications of transnasal TEE using ICE probes.

Author	Year	Study type	No.	Probe (head width)	Plane view	Type of anesthesia	Use of nasal Trumpet/Conduit	Success of nasal intubation	Adverse outcomes	Patient comfort	Image quality (Rated by clinicians)
Tallarico et al.17	2005	Case report	1	Siemens AcuNav (3.3 mm)	Mono	None	No	100% (single case)	None	Well tolerated	Adequate
Tallarico et al. [18].	2005	Case report	1	Siemens AcuNav (3.3 mm)	Mono	None	No	100% (single case)	None	Well tolerated	Adequate
Tallarico et al. [19].	2006	Case report	1	Siemens AcuNav (3.3 mm)	Mono	None	No	100% (single case)	None	Well tolerated	Adequate
Greco et al. [21].	2008	Prospective study	20	Siemens AcuNav (3.3 mm)	Mono	None	No	100%	None	Well tolerated	Acceptable/good in all cases
Schuster et al. [22].	2010	Case report	2	Siemens AcuNav (3.3 mm)	mono	None	No	100% (two cases)	None	Well tolerated	Adequate
Mitchell‐Heggs et al. [20].	2010	Prospective study	79	Siemens AcuNav (3.3 mm)	mono	None	No	100%	None	Well tolerated	Satisfactory in all cases

In 2006, Tallarico et al. [19], reported a case of transnasal TEE using a 10‐French (3.3 mm) Siemens AcuNav ICE catheter (monoplane, 5.5–10 MHz frequency range, 50 transducer elements) in a 42‐year‐old man with recurrent cryptogenic stroke. Conventional TEE identified an atrial septal aneurysm and PFO, and the patient subsequently underwent percutaneous PFO closure. To avoid general anesthesia and additional vascular access, the ICE probe was inserted transnasally without topical or general anesthesia. Image quality was sufficient to visualize the atrial septal anatomy, detect right‐to‐left shunting, and guide device deployment. Follow‐up TEE confirmed appropriate device positioning, and no procedural complications were reported.

Greco et al. [21], expanded on this approach in 2008 arguing that PFO closure is often unnecessarily prolonged by conventional TEE and the requirement for general anesthesia. They employed the same 10‐French AcuNav ICE catheter in 20 patients with ischemic stroke and PFO confirmed on prior TEE. All patients were receiving dual antiplatelet therapy. The probe was inserted transnasally without sedation, and patients remained awake and communicative throughout the procedure. All procedures were completed successfully, with high patient satisfaction and good tolerability. Similarly, Schuster et al. [22], described two additional cases in which transnasal TEE using the AcuNav ICE probe facilitated atrial fibrillation radiofrequency ablation. Both procedures were completed successfully with acceptable procedural durations.

The largest experience with ICE‐based transnasal TEE was reported by Mitchell‐Heggs et al. [20] in 2010, involving 79 patients undergoing atrial fibrillation ablation. All patients were on uninterrupted anticoagulation and had undergone conventional pre‐procedural TEE. A 10‐French AcuNav probe was inserted transnasally without sedation, and imaging focused on the atrial septum during transseptal puncture. The probe was withdrawn after successful left atrial access. All patients tolerated the procedure well, and image quality was considered adequate in every case. Notably, transnasal TEE identified three PFOs that had been missed on prior conventional TEE. The authors concluded that this technique offers a safe, well‐tolerated, sedation‐free, and potentially lower‐cost alternative to conventional TEE.

Despite demonstrating proof of concept, these studies were limited by suboptimal image quality due to outdated ICE probe technology, inconsistent probe‐esophageal contact [21], difficulty maneuvering the ICE probe following insertion [21], and traumatic bleeding [36]. Therefore, further technological innovation is required to overcome these limitations and fully realize the potential of transnasal TEE.

## 3 Discussion and Future Directions

A summary of published reports evaluating transnasal transesophageal TEE is provided in Tables 2, 3, 4, corresponding to mini probes, pediatric micro probes, and ICE probes, respectively. Collectively, these studies suggest that transnasal TEE represents a feasible alternative imaging modality in several clinical scenarios.

First, transnasal TEE may be particularly useful when conventional TEE is contraindicated. Contraindications may arise from anatomic limitations (oral lesions, severe trismus), patient intolerance or preference, or an elevated risk associated with sedation or general anesthesia. In such cases, the use of a smaller‐caliber probe inserted via the nasal route may provide diagnostic information comparable to conventional TEE. Recently, the DIONISO study (Intracardiac Echocardiographic Probe Used via Transesophageal to Guide LAA Occlusion) evaluated the feasibility of using the Acuson AcuNav 8F ICE probe, inserted transorally, to provide procedural guidance during LAA occlusion (LAAO) [37]. This study confirmed that a micro‐caliber transducer can provide sufficient visualization to support routine LAAO interventions. Building on this, a 2025 pooled analysis by Laterra et al. integrated data from the FLXiEST and DIONISO registries, utilizing 1:1 propensity score matching to compare the efficacy of gold‐standard TEE against transesophageal ICE (TE‐ICE) [38]. Their results demonstrated equivalent procedural success rates between the two modalities. These findings suggest that TE‐ICE can effectively reduce the clinical dependence on general anesthesia without compromising outcomes. Perhaps most significantly, the study validated that the significantly smaller footprint of an ICE probe does not preclude the high‐fidelity imaging required for complex structural heart interventions when imaging from the esophagus.

Second, transnasal TEE may be advantageous when rapid or serial assessment of cardiac structures is required and TTE is non‐diagnostic. This is commonly encountered in patients with recent sternotomy, severe obesity, advanced pulmonary disease, trauma, or in critically ill patients. In postoperative and intensive care unit (ICU) settings, TEE has demonstrated utility in evaluating hemodynamic instability, either independently or in conjunction with invasive hemodynamic monitoring [39, 40, 41, 42, 43]. However, conventional TEE is associated with procedural risks, and although major complications are uncommon, they can include significant bleeding, esophageal perforation [9], and hemodynamic compromise [9, 11], risks that may be amplified in critically ill patients requiring repeated examinations.

In an effort to improve safety and usability in this population, IMACOR (New York, NY, USA) [7] developed a transoral monoplane microprobe (ClariTEE), measuring 5.5 mm at the imaging head, which has received U.S. Food and Drug Administration approval for indwelling use of up to 72 h for hemodynamic monitoring. Following insertion, the probe handle can be detached, allowing the probe to remain in situ for intermittent, on‐demand imaging while the ultrasound system is used for other patients. This approach represents an innovative alternative to full‐size or pediatric TEE probes and has demonstrated clinical utility in the ICU setting [44]. However, inherent technological limitations, most notably the absence of multiplane imaging, restrict its broader application as a replacement for conventional TEE in perioperative, outpatient, and non‐ICU inpatient settings.

The concept of a “leave‐in” TEE probe is nonetheless compelling and aligns well with the needs of critically ill patients, particularly in those with poor transthoracic windows (i.e.post‐sternotomy). Continued advancements in probe technology, including multiplane capability, three‐dimensional imaging, and improved spatial resolution, could substantially expand the clinical utility of this approach.

Unsedated transnasal TEE allows patients to remain physiologically active during imaging. This enables dynamic assessment of mitral valve function, including exercise‐based interrogation (e.g., supine bicycle testing), which may reveal clinically significant mitral regurgitation or stenosis that could be underestimated in sedated patients under resting conditions [14, 15, 16].

Another important application involves evaluation for patent foramen ovale (PFO) in patients with cryptogenic stroke. Approximately 300,000 individuals experience cryptogenic stroke annually, and up to 40% are found to have a PFO [45, 46]. Conventional TEE is frequently used for PFO assessment [47]; however, sedation and anesthesia reduce intracardiac pressure gradients and preclude effective performance of a Valsalva maneuver, contributing to missed or misclassified diagnoses in up to 10% of cases [48]. In contrast, transnasal TEE permits evaluation in an awake, unsedated patient who can actively perform provocative maneuvers, thereby improving diagnostic sensitivity.

Recent advances in probe technology further support the feasibility of transnasal TEE. Both Philips (X11‐4t) and GE (9VT‐D) have introduced updated pediatric TEE probes with more than 2000 transducer elements and imaging capabilities approaching those of adult probes. Nevertheless, with imaging head widths of approximately 11 mm, these probes are unlikely to be suitable for transnasal insertion despite potentially reducing sedation requirements when used transorally.

Conversely, several manufacturers including Siemens (Volume ICE), Philips (VeriSight), and Johnson & Johnson Biosense Webster (NuVision), have developed U.S. Food and Drug Administration approved, three‐dimensional intracardiac echocardiography (ICE) catheters with diameters ranging from 3.0–4.2 mm. The VeriSight (3.0 mm) and NuVision (3.3 mm) systems, in particular, offer multiplane imaging and functionality that closely resemble conventional TEE. Adaptation of these probes for transnasal transesophageal use could allow for comprehensive, sedation‐free TEE studies with minimal patient discomfort. However, widespread adoption will require addressing economic and logistical barriers, as current 3D ICE probes are costly and designed for single‐use intravascular applications.

From a technical standpoint, miniaturized probes now possess many of the imaging capabilities as standard adult TEE systems. For transnasal TEE to gain broader acceptance, patient comfort must remain a central consideration. Evidence from the endoscopy literature consistently demonstrates superior tolerance and patient satisfaction with transnasal compared with transoral endoscopy, in both sedated and unsedated settings [49, 50, 51]. Smaller‐diameter probes, particularly those less than 5.3 mm, are associated with higher success rates and easier nasal insertion than slightly larger devices [52]. Additionally, as demonstrated in several studies reviewed herein, the use of a protective transnasal conduit or nasal trumpet may reduce mucosal trauma, improve patient comfort during probe manipulation, and potentially make a leave‐in probe more feasible.

## 4 Limitations

This review is constrained by the limited body of available clinical evidence, much of which reflects earlier technological constraints of available ultrasound probes. Most included studies were feasibility‐focused, with few direct head‐to‐head comparisons against conventional TEE and no randomized study designs, with most studies lacking advanced statistical methods such as propensity score matching or inverse probability weighting. Additionally, a substantial proportion of the literature predates 2010, likely reflecting a period of technological stagnation, as the Philips S8‐3t microprobe, introduced in 2004, lacked a true successor until the X11‐4t received FDA approval in early 2024. Similarly, advancements in intracardiac echocardiography lagged, with the original AcuNav ICE probes (introduced in 2000) only recently being supplanted by newer‐generation devices capable of multiplane and three‐dimensional imaging.

## 5 Conclusion

In conclusion, transnasal TEE represents a viable and complimentary addition to conventional TEE in patients with anatomic or hemodynamic contraindications, in those at elevated risk from sedation, and in clinical scenarios that may benefit from repeated or continuous cardiac assessment. While it is not intended to replace conventional TEE as a primary diagnostic tool, its development addresses specific unmet needs in specialized cardiac care scenarios. Optimization of a miniaturized, high‐resolution probe, whether an adapted pediatric TEE or ICE‐derived technology, capable of transnasal esophageal insertion has the potential to eliminate the need for sedation, reduce procedural risk, expand access, and provide more physiologically relevant diagnostic information. Further investigation is needed to evaluate the feasibility, performance, and cost‐effectiveness of emerging ultrasound probe technologies in this evolving clinical application.

## Funding

This work was supported by the National Heart, Lung, and Blood Institute [R33HL171790‐02].

## Ethics Statement

Not applicable.

## Patient/Participant Consent statement

No patient or participant consent was required for this review article.

## Conflicts of Interest

The authors declare no conflicts of interest. https://doi.org/10.13039/100000050
